# Stopping exploitation: Properly remunerating healthcare workers for risk in the COVID‐19 pandemic

**DOI:** 10.1111/bioe.12845

**Published:** 2021-02-07

**Authors:** Alberto Giubilini, Julian Savulescu

**Affiliations:** ^1^ Oxford Uehiro Centre for Practical Ethics University of Oxford Oxford United Kingdom of Great Britain and Northern Ireland; ^2^ Oxford Uehiro Centre for Practical Ethics and Wellcome Centre for Ethics and Humanities University of Oxford Oxford UK; ^3^ Visiting Professorial Fellow in Biomedical Ethics Murdoch Childrens Research Institute Melbourne Australia; ^4^ Distinguished Visiting International Professorship in Law University of Melbourne Melbourne Australia

**Keywords:** coercion, COVID‐19, exploitation, healthcare workers, incentives, payment for risk

## Abstract

We argue that we should provide extra payment not only for extra time worked but also for the extra risks healthcare workers (and those working in healthcare settings) incur while caring for COVID‐19 patients—and more generally when caring for patients poses them at significantly higher risks than normal. We argue that the extra payment is warranted regardless of whether healthcare workers have a professional obligation to provide such risky healthcare. Payment for risk would meet four essential ethical requirements. First, assuming healthcare workers do not have a professional obligation to take on themselves the risks, payments in the form of incentives would preserve autonomy in deciding what risks to take on oneself. Second, even assuming that healthcare workers do have a professional obligation to take on themselves the risks, payments for risk would create fair working conditions by avoiding exploitation. Third, payments for risk would make it more likely that public healthcare systems can discharge their institutional responsibility to provide healthcare in circumstances where healthcare workers may otherwise (perhaps legitimately) opt out. Fourth, payments for risk would guarantee an efficient healthcare system in pandemic situations. Finally, we address two likely objections that some might raise against our proposal, particularly with regard to incentives, namely that such payments or incentives can themselves be coercive and that they represent a form of undue inducement.

## 1 INTRODUCTION

A few days after the opening of the new Nightingale Hospital Yorkshire in April 2020, the UK Health Secretary Matt Hancock declared: ‘Whatever happens, we will make sure the NHS [the UK National Health Service] is there if you need it’. The hospital facility was quickly set up specifically to treat COVID‐19 patients in the face of shortage of intensive care unit (ICU) beds in other hospitals in the UK.

To keep such promises, Governments need not only healthcare facilities, but enough healthcare staff, as well as non‐healthcare staff working in healthcare facilities (e.g. janitors, cooks, and so on). However, some healthcare workers treating or caring for COVID‐19 patients, as well as some of the non‐healthcare staff, are exposed to significantly higher risks than in normal times. Simply asking these workers to take on such risks in order to allow a public healthcare system to discharge its institutional responsibility is ethically problematic. A few weeks after the hospital opening, the news came out that it had to turn away some COVID‐19 patients because of lack of staff, even though the facility itself did have space and ICU units to take more patients. The shortage was due to staff being employed in other hospitals, and not to their refusal to operate under exceptionally risky circumstances. Actually, NHS workers have taken on themselves a significant amount of additional risks to treat COVID‐19 patients. However, the problem of shortage of staff that emerged in the Nightingale Hospital case would be exacerbated and would likely present itself elsewhere if at some point healthcare workers are no longer willing to take on themselves those additional risks.

This is not an unrealistic prospect and something healthcare workers and non‐healthcare workers in healthcare settings might be ethically justified to do. Indeed, various healthcare workers associations have expressed their unease with the level of risk NHS staff are exposed to. For instance, in the UK as well as in other countries, one main issue especially at the beginning of the pandemic has been the shortage of personal protective equipment (PPE). The Doctors’ Association UK and the Royal College of Nurses warned that if risk would not be minimized, some of their members might soon decide to opt out of providing certain services.^1^ Even with PPE, though, the risks for healthcare workers would be higher than in normal times. If such risk is not adequately acknowledged through the payment that healthcare workers receive for providing their services, such workers would have legitimate grounds for opting out of riskier tasks. There would be no legitimate grounds to coerce them through threats of penalties. Doing that would be a form of exploitation, given their vulnerable bargaining position.

How can we ensure that workers in healthcare settings are treated fairly, not exploited, and that continuing to provide healthcare is a reasonable option for them, rather than the result of coercion?

In this paper, we argue that payment for the additional risks should be provided to frontline healthcare workers during the COVID‐19 pandemic. There is an ongoing debate on the boundaries of healthcare workers’ responsibilities during pandemics in general,^2^ and during the COVID‐19 pandemic specifically.^3^ One of the central questions is whether the level of risk involved falls within healthcare workers’ professional responsibilities, or whether it is supererogatory. The question is normally taken to be relevant to determine what, if anything, healthcare workers are owed in return for the service they provide under risky circumstances. Here, we want to suggest that healthcare workers should be paid for the additional risks they are taking during a pandemic like the COVID‐19 one quite independently of whether such risks fall within their professional responsibilities.

More precisely, we argue that payment for the extra risks is due to them on each of the following three different assumptions. The first one is that they do not have a professional obligation to take on themselves the additional risks entailed by treating or caring for COVID‐19 patients. The second is that they do have such an obligation, because that is what they sign up to when they enter the profession. The third is that they have a prima facie professional obligation to take on themselves such risks, but that this obligation does not apply if reasonable measures are not taken to minimize risks—most notably, adequate PPE supply. The ethical justification for the additional payment is different in each of these three scenarios, but we argue that it is very strong in all cases.

Not only would such payment ensure that the staff needed to deliver essential healthcare remain in the workforce. Also, it would ensure it for the right reasons. That is, healthcare workers would be treated fairly and not exploited, instead of being coerced to take on additional risks under unfair arrangements.

Importantly, in this paper we are focussing mainly on healthcare professionals such as doctors, nurses and allied health professionals, rather than non‐healthcare staff in healthcare facilities. The reason is that healthcare professionals might have a professional obligation to take on additional risks and burdens, so their case is more ethically problematic than the case of those who probably do not have such professional obligations (e.g. janitors or cooking staff). If the case for payments can be made for healthcare workers in spite of the possibility that they have such a professional obligation, then the same arguments would also apply a fortiori to staff in healthcare facilities who are also subject to significant additional risks without the corresponding professional obligations.

Also, the same arguments would apply to workers in other professions that, for various reasons, share some of the same risks and the same morally relevant features as the healthcare profession. These include, for instance, those whose work requires them to be in close proximity with healthcare workers (say, transit workers who bring high‐risk workers to their job) or those who do other jobs that are deemed ‘essential’ (say, garbage collectors) and who are also exposed to significantly increased risks during a pandemic (for example because trash can transmit viruses). Again, if our argument applies to healthcare workers who might have a professional obligation to take on additional risks, it applies a fortiori to such groups who might not have the equivalent obligation. As often is the case, though, consistency comes with a price, for instance in this case the risk of an increasing number of workers claiming increasingly large amounts of money in the name of equal treatment of all professions. This might require drawing a line at some point to separate level of risk and kinds of job that warrant payments for additional risk from those that do not. Some level of arbitrariness might be inevitable at some point. We will not address this problem here. We think that the fact that our argument might in practice require some level of arbitrariness if applied consistently to other professions, while certainly problematic, does not represent a fatal objection to it. Policies often draw arbitrary lines to address practical problems—it is not ideal, but is often unavoidable. But we do acknowledge that further reflection on what levels or risks and what jobs are eligible for additional payments for increased risks would be necessary, in order to identify criteria that are as ethically justifiable as possible rather than arbitrary.

## 2 RIGHT TO HEALTHCARE AND PROFESSIONAL RESPONSIBILITIES: A POSSIBLE TENSION DURING PANDEMICS

Throughout the first wave of this COVID‐19 emergency, most people in the UK clapped at their doorstep on Thursdays every week at the NHS ‘heroes’ that provide essential healthcare in challenging circumstances. Praising NHS workers is obviously warranted, considering the risks for their health and indeed their life that they are taking in order to preserve the efficiency of public healthcare. According to a report of the UK Office for National Statistics released at the end of June 2020, men healthcare workers had higher rates of deaths related to COVID‐19 than members of the general population.^4^

At the beginning of the COVID‐19 outbreak, residents in a retirement home in Madrid were abandoned and some were left to die in their beds.^5^ The home’s staff left after COVID‐19 was detected in the facility. The risks of being exposed to the virus were considered too high and not part of the staff’s professional responsibilities. We cannot exclude that the same shortage of staff might occur in hospitals where COVID‐19 patients are treated, if healthcare workers are not adequately paid for the risks they are taking. Even if they do have a professional responsibility to care for patients, which workers in care home might not have, such responsibility should be reflected in their payment. If the working conditions are unfair and exploitative, in the long term they might legitimately opt out of providing their services.

Here, we will assume that people have a right to receive adequate healthcare, especially in public healthcare systems. We will assume that the same right exists when they are infected with some life threatening and highly contagious virus, like COVID‐19. As we shall also mention, this is not as obvious as it might seem and there can be plausible arguments against the idea of a right to receive life saving healthcare in emergency situations and in conditions of limited resources. But *if* people have this right, a public healthcare system has the responsibility to guarantee adequate healthcare to everyone, consistently with fair allocation of scarce healthcare resources. However, individual healthcare workers might not have an obligation to deliver healthcare in all circumstances, including when the risk of doing so is not reflected in the payment they receive.

## 3 DO HEALTHCARE WORKERS HAVE AN OBLIGATION TO TAKE ON THEMSELVES THE RISKS ASSOCIATED WITH COVID‐19?

Healthcare workers do have a special positive duty to treat and care for sick patients. The duty is ‘special’ in the sense that it falls on healthcare workers in virtue of their professional obligations.^6^ And it is a positive duty in the sense that it requires its bearers to actively do something to assist a patient in need. Special positive duties typically require their bearers to take on more risks than would normally be expected of other people. But there are limits to such duties, of course.

There are different ethical grounds upon which professional obligations of healthcare workers can be based. Malm and colleagues (2008)^7^ identified five: expressed consent when taking up the job, implied consent, the special training that healthcare workers go through, duties of reciprocity of healthcare workers towards society (as part of a ‘social contract’ view), and obligations sanctioned by professional oaths and codes. According to them, none of these is sufficiently strong to ground an ethical or professional obligation to provide healthcare services involving *very large* risks, including the risk of being infected with a serious infectious disease like swine influenza or SARS. Consider the following analogy. A lifeguard ought to take on themselves more risks than a normal bystander on the beach in order to save someone drowning in the ocean. However, lifeguards are not required to take on themselves *whatever* risk is greater than the risk a normal bystander would be expected to take. For example, a lifeguard is probably not expected to rescue a swimmer from the mouth of a great white shark. It might be praiseworthy if they do that, of course, but it cannot be expected. According to this first view, requiring healthcare workers to take on themselves the additional risks entailed by a pandemic of a severe disease like SARS or influenza is like requiring a lifeguard to save a swimmer from the mouth of a great white shark.^8^

Others have argued for the opposite view, namely that healthcare workers do have an obligation to take on significant additional risks during pandemics, in virtue of the social contract between healthcare workers and society and of their special skills.^9^ This was also the approach often adopted when the problem of healthcare workers’ duties to take on themselves significant risks posed by infectious diseases started to be discussed in the ’90s, with regard to treatment and care of HIV patients. In that case, many thought that healthcare workers did have a duty to care, even at the cost of some additional risks for their health and life. Refusal to treat an HIV patient was often seen as expressing some form of prejudice against HIV positive people. Thus, for example, the American Nursing Association's official position on this matter was that ‘[n]ursing is resolute in its position that care should be delivered without prejudice, and it makes no allowance for use of the client's personal attributes, socioeconomic or health status as grounds for discrimination’.^10^ The *American College of Physicians ethics manual* stated that ‘the denial of appropriate care to a class of patients for any reason, *including disease state*, is unethical’^11^ (emphasis added). These phrasings suggest that the question about personal risks was very much intertwined, and indeed confused, with the issue of discrimination of certain categories of people.

However, even when the risk of discrimination is absent, such as in the case of treating COVID‐19 patients, some form of social contract between healthcare workers and the collective might still imply that healthcare workers do have a duty to treat in spite of the high risks. For instance, one consideration in favour of this view is that what matters for determining acceptable levels of risk is not only the actual risks involved in different scenarios, but also whether the scenarios could reasonably have been foreseen when someone took up a job. Now, it is true that the circumstances of the COVID‐19 pandemic are very exceptional. However, the risk of a global pandemic of a serious infectious disease has always been looming large in recent decades and through much of human history. Even if most countries were clearly unprepared to tackle the pandemic, it is not too surprising, and it should not be too surprising to healthcare workers, that something like the COVID‐19 outbreak occurred. The 2003 SARS epidemic, the 2009 swine flu pandemic, and the 2014 Ebola epidemic are only examples of how frequent and devastating infectious disease outbreaks can be. The Black Death killed one third of the European population—about 25 million people—over a 5‐year period, but the total number of deaths caused by the bubonic plague over the next two centuries is in the hundreds of millions.^12^ While COVID‐19 is having far worse effects at the global level than the recent epidemics, it is hardly a surprise. Actually, its effects could have been much worse and closer to the ones of the bubonic plague. Various experts and well‐known philanthropists supporting public health causes, including Bill Gates, have been warning for years about the risks that such a disease could emerge any time soon.^13^ We should be prepared for other and perhaps more dangerous viruses posing serious global health threats in the future. Those entering the healthcare professions would need to be aware of this risk.

However, whether or not we think that healthcare workers have duties to take on significant extra risks during pandemics, assessing such duties is not as straightforward as the reference to clear principles and theories—e.g. the ‘social contract’—might suggest. What responsibilities healthcare workers ultimately have also depends on how much consideration we want to give to other principles that can conflict with the duty of care, such as responsibilities towards one’s own family and towards other patients.^14^

Between these two camps, we can identify a third view that more specifically pertains to discussion of responsibilities during the COVID‐19 pandemic. This view takes into account the specific circumstances under which healthcare workers have often found themselves operating during this pandemic, most notably the lack of PPE. Given this situation, some have argued that even if healthcare workers are normally expected to take on the additional risks involved with dangerous infectious diseases, they do not have a professional obligation to take on themselves the significant additional risks entailed by this pandemic and that are created precisely by the lack of PPE.^15^ The additional risk has been made even greater precisely by the failure of governments to take reasonable steps to minimize it through stockpiling enough PPE during non‐pandemic times. Many countries, including the UK, were in this respect unprepared for a pandemic.

We do not take a stand on these different positions. It seems reasonable to claim that lack of PPE does weaken the case for a professional obligation to treat COVID‐19 patients in a way that exposes healthcare workers to significant risks. In any case, we argue that they should be paid for the additional risks that taking care of COVID‐19 patients entail under each of the three possible interpretations of professional obligations we have just provided.

## 4 EXTRA PAYMENT FOR HEALTHCARE WORKERS AT HIGHER RISK

Before arguing that such payments are fair, let us briefly explain why the issue is both practically and ethically important.

If healthcare workers are not fairly compensated for the risks, they have a legitimate claim to opt out of providing healthcare. The reason is simply that people do not have a moral or professional obligation to work under (too) unfair arrangements. Acting on this legitimate claim would jeopardize healthcare systems’ capacity to fulfil their responsibility to deliver adequate healthcare. We assume here we do not want to give up on a right to healthcare in pandemic situations. Admittedly, the idea that we should give up on the concept of universal and inalienable right to healthcare is not inconceivable. Giving up such a right during a pandemic like COVID‐19 would mean leaving people to die in their homes. It might entail providing just the basic assistance for palliative care that does not require trained staff assistance for its administration. Or it might mean providing medical assistance to dying. However, we are not going to explore these options here. If we think these are not viable options and that people have a right to receive healthcare aimed at saving their lives whenever reasonably possible, then we need functioning healthcare systems even during a pandemic. That is, we need healthcare workers to take on extra risks.

At the same time, we assume that it is not an option to exploit healthcare workers by forcing them to take on themselves very large risks for which they are not adequately paid. Forcing them would mean making their employment contingent upon accepting to work under unfair arrangements. Actually, our arguments would apply even if we accepted the proposal of limiting the amount of healthcare due to patients in crisis situations. Even with fewer patients to treat, healthcare workers would still be subject to additional risks, to the extent that the increased risk is not only a function of the number of infected patients to treat, but also of the severity and infectiousness of the virus in question—there will always be at least *some* infected patients to treat in a pandemic context.

The question is whether not being paid for the additional risks entailed by caring for COVID‐19 patients is exploitative, and therefore unfair, which would provide legitimate grounds for opting out. Here, we define exploitation simply as taking unfair advantage of someone who is in a weaker bargaining position^16^ in virtue of which they cannot reasonably refuse unfair offers. For example, not paying enough someone for the risks they take is exploitative if the person does not have enough bargaining power to ask for and obtain adequate payment. Exploitation typically trades on background injustice. A person would not accept an exploitative offer were it not for existing injustice or unfairness. Let us consider the three alternatives mentioned above.

First, suppose that healthcare workers do *not* have a professional or contractual duty to take on all the risks entailed by treating infectious diseases. In this case, opting out of such jobs should be an autonomous choice. That is, there should be no penalty attached, such as losing one’s job or having one’s salary cut. Forcing healthcare workers to choose between the alternatives of taking on the risks or facing some penalty would be a form of coercion, at least if we define ‘coercion’ in terms of reducing the range of options reasonably available to a person in a way that threatens the rights of that person.^17^ Coercion restricts a person’s options in a way that either harms that person or removes the option the person would autonomously prefer. We assume people should not be coerced into taking on risks that they are not supposed, expected, or ethically required to take. However, if we give them the option to withdraw from risky services, it is very likely that at least some would choose this option. It would be the most rational thing to do from a self‐interested, prudential perspective. It might even be the most ethical thing to do, if we consider their responsibilities towards themselves and their dependents,^18^ or their professional obligations towards other patients. However, if they do the rational thing, then the healthcare system might not be able to guarantee that universal right to healthcare we assumed above. The only way to keep the choice genuinely autonomous and to guarantee enough people autonomously choose risking their life is to make risk‐taking a reasonable option. Paying people enough extra money to take on the extra risks would serve both purposes. For example, we could create a scheme that offers incremental incentives on the basis of the level of additional risks.

Offering people money in return for risk that they take on themselves for the benefit of others, and which they cannot reasonably be expected to take, is already being discussed with regard to controlled human infection model (CHIM) studies, or ‘challenge studies’. These are trials where new treatments or vaccines are tested on research participants who have been purposely infected. Large enough incentives would allow the recruitment of enough participants to make the studies effective and to avoid exploiting vulnerable people who would volunteer for disproportionately small amounts of money.^19^ No one can simply be *expected* to volunteer as a participant in a CHIM study, given that there are some risks involved and that there usually is no benefit to the participants. If the collective is to benefit from the studies, we need to make sure that the benefit does not result from either coercion or exploitation. The same applies to healthcare workers who are forced to take on risks for insufficient money. Healthcare workers’ normal salary does not take such risks into account: riskier roles in healthcare are normally not paid more *because of* the risks. Hence, requiring them to take on certain risks would be exploitative, according to our definition of ‘exploitation’. Risk should be acknowledged. If there is a benefit to the collective or to individual people (i.e. sick patients) resulting from someone taking on risks that they are not ethically or professionally required to take, it seems fair that this risk taking be voluntary—because it cannot be simply ‘expected’–, properly encouraged—because we do need people taking on such risks—, and adequately paid for—, because we do not want to exploit those who would accept small amounts of money for large risks out of need.

The second alternative is that healthcare workers do not have a duty to take on the extra risks when they lack adequate PPE. In such case, the same consideration as above applies. We cannot simply expect them to take on such risks, if they could have easily been prevented. During this pandemic, shortage of PPE has often been due not to lack of current financial investment, but to a global shortage of supply and distribution shortcomings due to past choices—this was the case in some countries (such as the UK) when the COVID‐19 pandemic started.^20^ Therefore, it seems fair that governments invest the money they would use to provide adequate PPE to healthcare workers to instead incentivize them to stay in the workforce. Governments, and therefore the collective, are blameworthy for creating the conditions that could make healthcare workers’ opt out legitimate. Therefore, having they the responsibility for adequate healthcare provision, they have a responsibility to redress this situation by investing resources in a way that incentivizes staying in the workforce despite the risks. This is the only way healthcare systems can discharge their responsibility to provide healthcare, given the circumstances.

Finally, suppose healthcare workers do have a professional responsibility to treat or care for COVID‐19 patients, because that is what they sign up to and because the lack of PPE does not make a significant enough difference to their obligations. Even in this case, the risk they are subject to should be fairly considered. The mere fact that something falls within one’s professional obligations does not mean that that it should not be acknowledged in the form of additional payments. When employees are required by their employers to work extra hours as overtime, for instance, they often have a professional or contractual obligation to do it, within certain limits. If there is extra work to be done, often employees cannot refuse. This is true for many professions. However, this does not mean that they should not be paid for the extra time they work. Actually, some have suggested precisely that healthcare workers treating or caring for COVID‐19 patients are owed some form of compensation because the emergency often requires them to work more hours and to work under more stressful conditions.^21^ But if extra hours should be paid for, so should extra risk. The reason is that in both cases workers are required to do something that, precisely because they might have professional obligations to do, they might not be in a position to refuse doing, but that represents a significant additional burden to them, compared to the burdens for which they are paid. Just as professionals can be expected to do some reasonable unpaid overtime, they can also be expected to take on some reasonable extra risks. But over this reasonable limit, they require extra compensation.

Even if they have a professional duty to take on significant risks, like significant overtime, unless the risk is already accounted for in their contracted salary, they should be paid for such risks. As far as we are aware, at the moment riskier healthcare jobs in a healthcare system like the NHS are not paid more in virtue of the risks involved. Healthcare workers might well have a professional obligation to take on such risks. But expecting them to do it for free, that is, for their contracted salary, is a form of exploitation. If their employer expects them to do so, it means that the alternative they have is between leaving their jobs and taking on risks not factored in in their salary. This expectation would put them in a very vulnerable bargaining position, which would make them easy targets for exploitation. Moreover, staying in the job under these conditions would likely be the result of coercion because the option of keeping their job under a fair arrangement would be removed. They would have to either take on extra risks for which they are not paid, or leave their job.

On the basis of all these considerations, we can identify four ethical principles that support extra payments for risks for healthcare workers dealing with COVID‐19 patients. These are autonomy, fairness, institutional responsibility, and expected utility. While respect for autonomy is only relevant in case we assume healthcare workers do not have a professional obligation to treat or care for COVID‐19 patients, the others apply even if they do have such an obligation.

*Autonomy*. Respecting healthcare workers’ autonomy to decide whether or not to take on themselves the additional risks is only relevant if we assume that they do not have a professional or contractual obligation to do so. Offering the option of the extra payment for the extra risk would allow healthcare workers to autonomously decide what level of risk to take on themselves beyond the risks that they are normally expected to take. While taking on additional risks for free is not an option for many, because it would rightly be perceived as unreasonable, payments (in the form of incentives) for such risks would make that option more reasonable, and therefore part of the range of options that a healthcare worker could reasonably choose. We should not use coercion—e.g. by threatening to fire them—if there is a less liberty restricting option.

Now, according to some philosophical views, large incentives can infringe upon autonomy and constitute a form of coercion.^22^ However, we do not think that offers can be coercive. We agree with the standard account of ‘coercion’ provided by Alan Wertheimer, both at the theoretical level^23^ and specifically with regard to incentives for risky activities that benefit society.^24^ As tempting as they can be, offers expand the range of options available to someone and therefore give them more choice, without harming or violating the rights of those who receive the offer. A necessary condition for a proposal to be coercive is that it reduces the range of options reasonably available to someone (and according to Wertheimer, even that is not enough, as options must be restricted in a way that threatens some right of a person in order for that person to be coerced). For instance, ‘your money or your life’ paradigmatically removes options and therefore is coercive, because you cannot keep both your money and your life, which is what of course you would normally choose. Offering incentives is the exact opposite of this.

The ethical issue that arises with incentives is not coercion, but exploitation.^25^ That is, the problem arises not if incentives are too large, but if incentives are too small and those with very weak bargaining power (for example, someone in a dire financial situation) would still be tempted to accept them. A very large incentive is difficult to refuse just because the goods it buys outweigh the harms or risks associated with it. But it is not coercive because the option of refusing it remains available without making the individual worse off. Offering a person a million dollars to relocate to a different job may be difficult to refuse, but it is not coercion. The person can keep the current job without being made worse off—in the same way as a healthcare worker refusing the incentive to take on additional risks would not be made worse off, as they can keep their current working conditions without the additional money and the additional risks. Not everything that makes an option difficult or unreasonable to refuse is coercive, at least according to the account of coercion we have defended above.

*Fairness*. Extra payment for extra risk would acknowledge the actual risks healthcare workers take on themselves, on top of those they normally take during everyday work. Taking on oneself such risks might not be beyond the ‘call of duty’ of the profession, but it might be beyond the call of contractual duty if healthcare workers’ normal salary does not already factor in the possibility of such risk taking. Forcing healthcare workers to work for normal salary despite the additional risk would be a form of exploitation, that is, of paying people less than they are owed by exploiting their vulnerable bargaining position. It is exactly the same as paying someone normal salary when they work extra hours. And the fact that the community needs healthcare workers to take on such risks suggests that the community owes them something in return, as a matter of reciprocity,^26^ which is another dimension of fairness. The way public money is normally spent to guarantee basic healthcare does not take into account such risks. Even assuming healthcare is a universal right, it does not come for free. We use public money to guarantee a certain level of public healthcare in normal times. In the same way, we should use public money to guarantee a certain level of public healthcare in exceptional times, even if that requires an exceptional amount of money.

*Institutional responsibility*. Extra payment for extra risk would allow a healthcare system to discharge its obligations to provide healthcare to those who have a right to it, when we cannot expect individual healthcare workers to make their contribution to healthcare delivery under normal salary arrangements. The way a healthcare system discharges its responsibility in this case is by making additional resources available to ensure risk taking is properly acknowledged. In this way, healthcare workers would not have legitimate grounds for opting out. Payments for risks would therefore likely preserve the capacity of healthcare systems to fulfil their institutional responsibility in emergency circumstances. It is important to emphasize that the responsibility in question is that of healthcare systems to provide healthcare, not that of individual healthcare workers to provide their services under unfair arrangements, no matter what professional obligations to care they have.

*Expected utility*. Related to the previous point, extra payment could allow healthcare systems to provide valuable services without ethical costs. By removing the ground for legitimate withdrawal from service, the efficiency of the healthcare system would likely be preserved without coercion and without exploitation. The system would also be more efficient because the payment for risks would likely give healthcare workers more motivation to do their job properly. After all, we do not want healthcare workers who do not want to be there. Importantly, the same considerations apply also where there is no current shortage of staff and therefore it might look like such payments are not needed. Considerations of expected utility require us to take into account the probability of negative outcomes occurring—in this case, the risk of sudden shortage of staff, as happened for example in the case of the care home in Madrid we mentioned at the beginning. Preventing such scenarios is an additional consideration in support of payments on the basis of maximization of expected utility.

## 5 MEETING TWO OBJECTIONS

We have argued above why we think offers of incentives or payments for risks are not coercive. However, suppose *for the sake of argument* that we acknowledge that incentives or payments for risks do entail some level of coercion. This could be the case if we endorsed some accounts of coercion^27^ merely in terms of presenting options that are unreasonable to refuse. Still, we do not think this would undermine our argument. Assuming we do want to guarantee a right to healthcare to COVID‐19 patients, the alternative to payment for risk would be to force healthcare workers to treat and care for patients under normal salary conditions, but with non‐normal levels of risks. That is, the alternative to our proposal would definitely be more coercive even if we assume, for the sake of argument, that our proposal is somewhat coercive.

Some would object that payments for risk represent undue inducement. Large payments would make risk taking more appealing. Some fear this could lead individuals to discount risks,^28^ which is problematic if we assume people should be put in conditions to make an autonomous choice whether or not to take the risks. We disagree. What counts as ‘undue’ is highly subjective. What matters is that individuals understand levels of risk and are put in the condition to make a genuinely autonomous decision about what risks to take. It is very unlikely that healthcare workers would not understand levels of risk. We might disagree with their risk assessment and think that we would not make the kinds of risk‐taking decisions some of them would make. But to say that their risk assessment is irrational or reflects mistaken assessment when extra payments are involved is very paternalistic.^29^ When people are competent, informed, and put in the condition to make autonomous decisions, paternalism is not ethically acceptable. In this specific case, nobody is better placed to assess the risks than healthcare workers.

## 6 CONCLUSION

We have argued that healthcare workers should receive extra payments for the significant additional risks involved in delivery of healthcare to COVID‐19 patients, even if they have a professional obligation to take such risks. Payments would be consistent with fair working conditions because they would acknowledge the extra risks healthcare workers are taking. They would allow healthcare systems to discharge their institutional responsibilities to provide healthcare. The case for such payments is even stronger if we assume healthcare workers do not have professional responsibilities to take on such risks. In such case, payments should take the form of incentives, which would preserve healthcare workers’ autonomy to decide what level of risks to take on themselves, when they cannot be expected to take on those risks. Ultimately, payments and incentives would prevent coercion and exploitation of healthcare workers and would benefit the collective by making it more likely that governments can discharge their responsibility to provide an adequate level of healthcare during the COVID‐19 crisis, or indeed during any future similar public health emergency. How much healthcare workers should be paid for risk is a large and open question, but payment for risk is standard through other areas of employment. Similar payments for similar risks would be a good place to start. Otherwise, we discriminate against healthcare workers.

## CONFLICT OF INTEREST

The authors declare no conflict of interest.

## Footnotes

^1^

Campbell, D., & Stewart, H. (2020, March 24). *Doctors threaten to quit NHS over shortage of protective kit*. The Guardian. https://www.theguardian.com/world/2020/mar/24/doctors‐threaten‐to‐quit‐over‐protective‐equipment‐shortage

^2^

e.g. Clark, C. (2005). In harm's way: AMA physicians and the duty to treat. *Journal of Medicine and Philosophy*, *30*(1), 65–87. 10.1080/03605310590907066; Malm, H., May, T., Francis, L. P., Omer, S. B., Salmon, D. A., & Hood, R. (2008). Ethics, pandemics, and the duty to treat. *The American Journal of Bioethics, 8*(8), 4–19. 10.1080/03605310590907066.

^3^

Dunn, M., Sheehan, M., Hordern, J., Turnham, H. L., & Wilkinson, D. (2020). ‘Your country needs you’: the ethics of allocating staff to high‐risk clinical roles in the management of patients with COVID‐19. *Journal of Medical Ethics*, *46*, 436–440; Johnson, S. B., & Butcher, F. (2021). Doctors during the COVID‐19 pandemic: What are their duties and what is owed to them? *Journal of Medical Ethics*, *47*, 12–15; McConnell, D. (2020). Balancing the duty to treat with the duty to family in the context of the COVID‐19 pandemic. *Journal of Medical Ethics*, *46*, 360–363; Schuklenk, U. (2020, April 1). Health care professionals are under no ethical obligation to treat COVID‐19 Patients. *JME Blog*. https://blogs.bmj.com/medical‐ethics/2020/04/01/health‐care‐professionals‐are‐under‐no‐ethical‐obligation‐to‐treat‐covid‐19‐patients/ 10.1136/medethics-2020-106284PMC724609232409625

^4^

Office for National Statistics. (2020). *Coronavirus (COVID‐19) related deaths by occupation, England and Wales: deaths registered between 9 March and 25 May 2020*. https://www.ons.gov.uk/peoplepopulationandcommunity/healthandsocialcare/causesofdeath/bulletins/coronaviruscovid19relateddeathsbyoccupationenglandandwales/deathsregisteredbetween9marchand25may2020/previous/v1

^5^

BBC. (2020, March 24). *Coronavirus: Spanish army finds care home residents ‘dead and abandoned’*. https://www.bbc.co.uk/news/world‐europe‐52014023

^6^

Sokol, D. K. (2006). Virulent epidemics and scope of healthcare workers' duty of care. *Emerging Infectious Diseases*, *12*(8), 1238–1241. 10.3201/eid1208.060360 PMC329123416965703

^7^

Malm et al., op. cit. note 2.

^8^

Ibid.

^9^

Clark, op. cit. note 2.

^10^

American Nurses Association (ANA). (1994). *Ethics and human rights position statements: Risk versus responsibility in providing nursing care*. Effective date: December 8, 1994, status: revised position statement, originated by ANA Committee on Ethics, 1986. Silver Spring, MD.

^11^

American College of Physicians. (1998). Ethics manual: Fourth edition. *Annals of Internal Medicine*, *128*(7), 576. 10.7326/0003-4819-128-7-199804010-00012 9518406

^12^

Britannica, The Editors of Encyclopaedia. (2020). “Black Death”. Encyclopedia Britannica, 9 Nov 2020, https://www.britannica.com/event/Black‐Death. Accessed 29 January 2021.

^13^

Hoffower, H. (2020, December 15). Bill Gates has been warning of a global health threat for years. Here are 12 people who seemingly predicted the coronavirus pandemic. Business Insider, https://www.businessinsider.com/people‐who‐seemingly‐predicted‐the‐coronavirus‐pandemic‐2020‐3?r=US&IR=T. Accessed 29 January 2021.

^14^

Sokol, op. cit. note 6; McConnell, op. cit. note 3.

^15^

Schuklenk, op. cit. note 3; Johnson & Butcher., op. cit. note 3.

^16^

Zwolinski, M., & Wertheimer, A. (2017). Exploitation. In E. N. Zalta (Ed.), *The Stanford encyclopedia of philosophy*, (Summer 2017 Edition). https://plato.stanford.edu/archives/sum2017/entries/exploitation/

^17^

Wertheimer, A. (1987/89). *Coercion*. Princeton University Press, pp. 30, 36–37; Wertheimer, A., & Miller, F. G. (2008). Payment for research participation: A coercive offer? *Journal of Medical Ethics, 34*, 389–392.10.1136/jme.2007.02185718448723

^18^

McConnell, op. cit. note 3.

^19^

Grimwade, O., Savulescu, J., Giubilini, A., Oakley, J., Osowicki, J., Pollard, A. J. & Nussberger, A. M. (2020). Payment in challenge studies: Ethics, attitudes and a new payment for risk model. *Journal of Medical Ethics*, *46*, 815–826; Anomaly, J., & Savulescu, J. (2019). Compensation for cures: Why we should pay a premium for participation in ‘challenge studies’. *Bioethics, 33*, 792–797.10.1136/medethics-2020-106438PMC771990032978306

^20^

Financial Times. (2020, April 1). Why the NHS is struggling to protect staff from Coronavirus. https://www.ft.com/content/18bf272b‐0b3c‐433c‐a484‐7f653c82ff1b

^21^

Johnson & Butcher, op. cit. note 3.

^22^

e.g. Feinberg, J. (1989). *The moral limits of the criminal law*. Oxford University Press.

^23^

Wertheimer, op. cit. note 17.

^24^

Wertheimer & Miller, op. cit. note 17.

^25^

Savulescu, J. (2001). The fiction of ‘undue inducement’. *The American Journal of Bioethics, 1*, 1–3.

^26^

Johnson & Butcher, op. cit. note 3.

^27^

e.g. Feinberg, op. cit. note 22; Frankfurt, H. (1988 [1973]). Coercion and moral responsibility. In *The importance of what we care about* (pp. 26–45). Cambridge University Press. First published in T. Honderich (Ed.), *Essays on freedom of action* (pp. 65–86). Routledge & Kegan Pau; Held, V. (1972). Coercion and coercive offers. In P. J. Roland & J. W. Chapman (Eds.), *Nomos XIV: Coercion* (pp. 49–62). Aldine‐Atherton Inc; Nozick, R. (1969). Coercion. In S. Morgenbesser, P. Suppes, & M. White (Eds.), *Philosophy, science, and method: Essays in honor of Ernest Nagel* (pp. 440–472). St. Martin’s Press.

^28^

Grady, C. (2001). Money for research participation: Does it jeopardize consent? *The American Journal of Bioethics*, *1*, 40–44.10.1162/15265160130016903111951886

^29^

Savulescu, op. cit. note 25.

